# Marek’s disease in chicken farms from Northwest Ethiopia: gross pathology, virus isolation, and molecular characterization

**DOI:** 10.1186/s12985-023-02003-4

**Published:** 2023-03-08

**Authors:** Mastewal Birhan, Esayas Gelaye, Saddam Mohammed Ibrahim, Nega Berhane, Takele Abayneh, Belayneh Getachew, Aragaw Zemene, Kassahun Birie, Getaw Deresse, Kassaye Adamu, Bereket Dessalegn, Abebe Tesfaye Gessese, Mebrie Zemene Kinde, Molalegne Bitew

**Affiliations:** 1 Institute of Biotechnology, University of Gondar, Gondar, Ethiopia; 2 College of Veterinary Medicine and Animal Sciences, University of Gondar, Gondar, Ethiopia; 3 National Veterinary Institute, Debrezeit, Ethiopia; 4 Bio and Emerging Technology Institute, Addis Ababa, Ethiopia

**Keywords:** Chickens, ICP4 gene, Marek’s disease, Marek’s disease virus, Molecular characterization, Northwest Ethiopia, Virus isolation

## Abstract

Marek’s disease virus (MDV) is a highly contagious, immunosuppressive, and oncogenic chicken pathogen causing marek’s disease (MD). In this outbreak-based study, 70 dual-purpose chickens that originated from poultry farms in Northwest Ethiopia and suspected of MD were sampled for pathological and virological study from January 2020 to June 2020. Clinically, affected chickens showed inappetence, dyspnea, depression, shrunken combs, and paralysis of legs, wings, and neck, and death. Pathologically, single or multiple greyish white to yellow tumor-like nodular lesions of various size were appreciated in visceral organs. In addition, splenomegaly, hepatomegaly, renomegaly, and sciatic nerve enlargement were observed. Twenty-seven (27) pooled clinical samples i.e. 7 pooled spleen samples and 20 pooled feathers samples were aseptically collected. Confluent monolayer of Chicken Embryo Fibroblast cells was inoculated with a suspension of pathological samples. Of this, MDV-suggestive cytopathic effects were recorded in 5 (71.42%) and 17 (85%) pooled spleen and feather samples respectively. Molecular confirmation of pathogenic MDV was conducted using conventional PCR amplifying 318 bp of *ICP4* gene of MDV-1, of which, 40.9% (9/22) tested positive. In addition, 5 PCR-positive samples from various farms were sequenced further confirming the identity of MDV. The *ICP4* partial gene sequences were submitted to GenBank with the following accession numbers: OP485106, OP485107, OP485108, OP485109, and OP485110. Comparative phylogenetics showed, two of the isolates from the same site, Metema, seem to be clonal complexes forming distinct cluster. The other three isolates, two from Merawi and one from Debretabor, appear to represent distinct genotypes although the isolate from Debretabor is closer to the Metema clonal complex. On the other hand, the isolates from Merawi appeared genetically far related to the rest of the 3 isolates and clustered with Indian MDV strains included in the analysis. This study presented the first molecular evidence of MDV in chicken farms from Northwest Ethiopia. Biosecurity measures should strictly be implemented to hinder the spread of the virus. Nationwide studies on molecular characteristics of MDV isolates, their pathotypes, and estimation of the economic impact associated with the disease may help justify production and use of MD vaccines within the country.

## Introduction

The quest to satisfy the ever-rising demand for protein food has led to the intensification and industrialization of poultry farming. Despite improving productivity of the sector, this also lend to a situation highly conducive to pathogen evolution as a result of cramped living conditions and shorter rearing periods. A typical example of this is marek’s disease virus (MDV), which is a pathogen of poultry that has evolved from a relatively harmless paralytic syndrome into a highly virulent pathogen [1] as a result of industrialization [2, 3].

Marek’s disease (MD) is a highly contagious, immunosuppressive [4], and lymphoproliferative disease of chickens [5] with a mortality rate reaching 100% [1, 6]. The causative agent, Gallid alphaherpes virus 2 (GaHV-2), is a dsDNA virus belonging to the genus *Mardivirus*, subfamily *Alphaherpesvirinae,* and family Herpesviridae [7]. Within the genus *Mardivirus,* are three closely related but distinct virus species. Gallid alphaherpes virus 2 (formerly serotype 1 MDV, MDV-1), consists of all known pathogenic strains, which vary in their pathogenic and oncogenic potential and being classified as mild (m), virulent (v), very virulent (vv), and very virulent plus (vv +) GaHV-2 [8, 9]. Gallid alphaherpes virus 3 (GaHV-3, formerly serotype 2 MDV), and Meleagrid alphaherpes virus 1 (MeHV-1, also called herpes virus of turkey (HVT) and formerly serotype 3 MDV) are avirulent strains isolated from chickens and turkey respectively [7].

The disease is characterized by general inflammation of peripheral nerves (polyneuritis) and development of solid tumors in multiple organs that originate from transformed T lymphocytes [10–12]. Two forms of the disease are well recognized, neural and visceral types [13], with 10–25% and above 70% mortality, respectively. In neural MD, the main clinical symptoms include a complete or partial paralysis of the neck, wings, and limbs. Such paralyses are mainly induced by lesions of the vagus, brachial, and sciatic plexuses that show enlargement and yellowish color on the surface. In visceral MD, the gross tumors can be observed in the gonads, liver, kidney, lung, heart, spleen, and proventriculus in larger sizes and higher numbers [14]. At necropsy, MD gross lesions are characterized by diffuse enlargement of the liver and the spleen, presence of lymphomas in liver, kidney, ovary, proventriculus, spleen, lungs, nerves, heart, skin, and atrophy of the bursa of Fabricius and thymus [15].

Diagnosis is based on isolation and identification of MDV from infected tissues. Virus isolation is usually by virus propagation in cell culture and identification/quantification by cytopathic changes (plaque formation) or identification of the infected cells by immunostaining [16]. Several PCR and real-time-based techniques have been developed for detection, as well as quantification, of the MDV genome of field and vaccine strains from blood, organ samples, and feather tips [17]. In addition, loop-mediated isothermal amplification (LAMP), has been presented as attractive alternative to the PCR-based methods. LAMP is a rapid technique that can be performed at a single temperature (60 °C to 65 °C) in a laboratory water bath or a dry heat block, and the results can be read with the naked eye [18].

Vaccination against MD denotes one of the most successful examples of protection against virally induced tumor. All the currently used vaccines are live vaccines derived from the three viral strains: the HVT FC126 strain [19], the GaHV-3 SB-1 strain [20], and the GaHV-2 CVI988/Rispens strain [21]. HVT and SB-1 vaccines are considered heterologous vaccines as they are derived from a different viral species than the target virus, while the Rispens vaccine is considered homologous because it is from the same viral species as the targeted virus.

Currently, MD has a global distribution with increasing reports of vaccination breaks and the emergence of more virulent pathotypes [22, 23] and is responsible for a massive economic burden [24]. Outbreaks of MD have been reported in different parts of Ethiopia. Lobago and Woldemeskel (2004) [25]have reported MD in a commercial poultry farm in Central Ethiopia on clinico-pathological criteria. Similarly, Duguma et al. (2005) [26] conducted serological and clinico-pathological investigation reporting a higher (97.9%) mortality of chickens due to MD. However, the first genetic confirmation of MDV-1 was reported by Demeke et al. (2017) [27]from commercial farms in Central Ethiopia. Furthermore, MDV has been isolated from outbreaks in different zones of Southwestern Ethiopia [28]. It is becoming clear that farms in the country need to consider MD prevention and control plans to avoid the drastic consequences of the disease. Despite the occurrences of disease outbreaks suggestive of MD in chickens in Northwest Ethiopia, there are no genetic evidences and official reports on the presence and circulation of MDV in the region. In this study we conducted a clinico-pathological and molecular investigation in MD-suspected outbreaks in commercial poultry farms in Northwest Ethiopia. Thus, this study provides the first molecular evidence of MDV-1 in chicken flocks in Northwest Ethiopia.

## Materials and methods

### Study area

The chicken farms addressed in this study are located in the Northwest part of the Amhara National Regional State (ANRS) of Ethiopia. The ANRS is located in the Northwestern part of the country between 9°20′ and 14°20′ North latitude and 36° 20′ and 40° 20′ East longitude. The region is organized into several administrative zones, of which, North Gondar, South Gondar, and West Gojjam zones were included in this study.
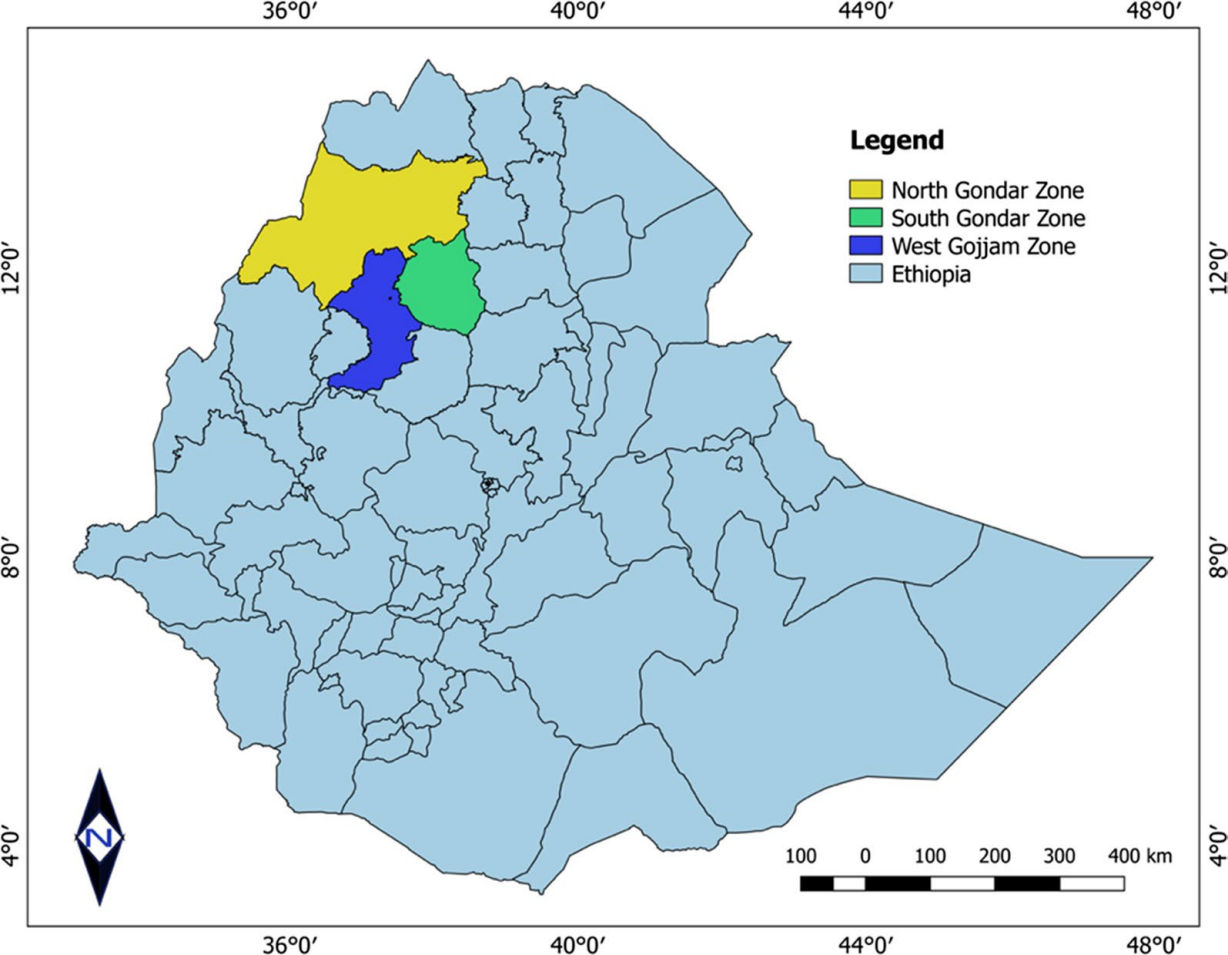


### Postmortem examination and sample collection

MD-suspected outbreaks were investigated from January 2020 to June 2020. Seventy (North Gondar = 35, South Gondar = 12, and West Gojjam = 23), dual-purpose chickens of all age, presumed to be morbid and dead due to MDV, were collected from various poultry farms, sacrificed by cervical dislocation, and necropsied for gross pathological examination in the Veterinary Pathology Laboratory of the College of Veterinary Medicine and Animal Sciences, University of Gondar. None of the flocks had a history of MD vaccination.

Macroscopic changes relevant to MD were recorded on major organs, nerves, and body cavities. Twenty-seven [27] pooled clinical samples i.e. spleen (*n* = 7, 3 pooled samples from North Gondar and 2 pooled samples from each of South Gondar and West Gojjam zones) and feathers follicles (*n* = 20, 6 pooled feather samples for North Gondar and 7 pooled samples from each of South Gondar and West Gojjam zones) were aseptically collected from chickens with postmortem lesions indicative of MD. The samples were transported in cryovials containing virus transporting media in an ice box into the Veterinary Virology Laboratory of the National Veterinary Institute (Debre zeit, Ethiopia) and preserved at − 20 °C until processed and analyzed for virus isolation according to the OIE [29].

### Virus isolation from spleen

First, spleen samples were minced into small pieces using sterile scissors and then grounded using sterile mortar and pestle. A sterile phosphate buffer saline (PBS) supplemented with penicillin (100 IU/ml) and streptomycin (1000 μg/ml) was used to prepare a 10% (w/v) suspension of spleen samples. The suspension in a sterile tube was centrifuged at 10,000 rpm for 10 min at 4 °C. Then, a confluent primary chicken embryo fibroblast (CEF) cells prepared from specific pathogen free eggs (VALO BioMedia, Germany) containing maintenance GMEM (Sigma) plus 2% bovine fetal calf serum (Gibco) was inoculated with 0.5 ml of the supernatant and incubated at 37 °C. Cultures were monitored daily for virus induced cytopathic effect (CPE) under an inverted microscope for 1 week [29]. Cultures that did not show CPE were blindly passage till third passages before declared negative. Samples revealing characteristic CPEs were considered positive and kept at − 20 °C for further molecular analysis.

### Virus isolation from feather follicles

Nine feather follicles from individual affected chicken were collected and processed according to Woźniakowski et al. [30]. A 10% (w/v) suspension of feather tips (about 5 mm long) or minced tracts of skin containing feather tips suspended in sucrose, phosphate, glutamate, and albumin/ethylenediamine tetra acetic acid (SPGA/EDTA) buffer was homogenized for 3–5 min and then sonicated for 2 min. The procedure for inoculation onto cell culture was the same as described above for the spleen samples.

### DNA extraction

DNA was extracted from tissue homogenates using DNeasy® Blood and Tissue Kit (Qiagen, Germany) following the manufacturer’s guide. The extracted DNA was eluted in 40 μL elution Buffer and kept at –20 °C until analysis.

### PCR detection of MDV-1

PCR was performed by amplifying the *ICP4* gene (major transcriptional regulatory protein) of MDV-1 using the primers: forward primer M1.1, 5ˊ-GGATCGCCCACCACGATTACTACC-3ˊ and reverse primer M1.8, 5ˊ-ACTGCCTCACACAACCTCATCTCC-3ˊ) as previously described by Kalyani et al. [17]. The primers were synthesized by VBC Biotech and purified by reverse phase high-performance liquid chromatography (Vienna, Austria). The PCR was conducted in a final reaction volume of 20 μL using 200 μL capacity thin wall PCR tube containing 10 X PCR buffer (Qiagen), 25 mM MgCl2, 200 nM of each of the four dNTPs, 0.5 pmol/μL of each primer, 1U Taq DNA polymerase, and 4 μL of templates DNA. The following PCR protocol was applied: an initial denaturation at 95 °C for 5 min, followed by 35 cycles of denaturation at 95 °C for 30 s, annealing at 58 °C for 30 s, and extension at 72 °C for 30 s, and final extension at 72 °C for 5 min. The PCR products were analyzed using 1.5% agarose gel stained with GelRed (Biotium, Inc). The gel was run at 120 V for 1 h on an electrophoresis apparatus (EC 2060, USA) and the bands were visualized in a UV trans-illuminator (UVI TEC, UK).

### Partial sequencing of *ICP4* gene of MDV-1

Amplified PCR products (*n* = 5) were purified using Wizard® SV Gel and PCR product purification kit (Promega, Germany) according to the instructions supplied by the manufacturer. Concentration of extracted total DNA was quantified using micro-volume spectrophotometer (Nanodrop 2000c, USA) and sequenced by LGC Genomics (Germany). The sequences were edited, and contig was formed using Vector NTI 11.5 software (Invitrogen). For comparative phylogenetic analysis, blastn was used to collect additional sequence data of *ICP4* gene from GenBank. All sequence analysis was performed in MEGA version 11 [31, 32]. Multiple sequence alignments were executed using MUSCLE program. Evolutionary relationship among the current isolates and isolates from other geographical areas was estimated based on phylogenetic trees constructed using the Maximum Likelihood with Kimura 2-parameter method with bootstrap replicates set at 1000.

## Results

### Outbreak description

**Fig. 1:**
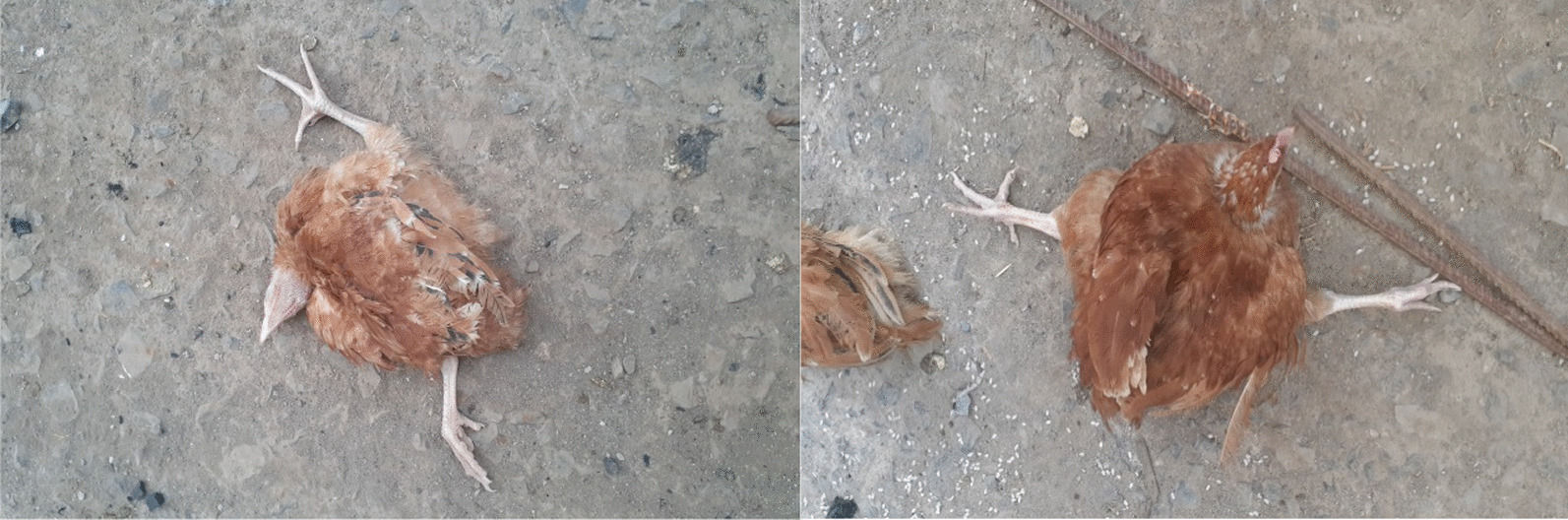
Chickens presumed to be affected by a marek’s disease and showing split paralysis and twisting of the head to the side

Affected birds showed inappetence, loss of weight, dyspnea, depression, shrunken combs, and paralysis of leg, wing, and neck. Chickens were found lying on the ground in a splay legged position, and struggling to move. In addition, dead birds were also evident during the outbreak investigation (Fig. 1).

### Gross pathology

**Fig. 2:**
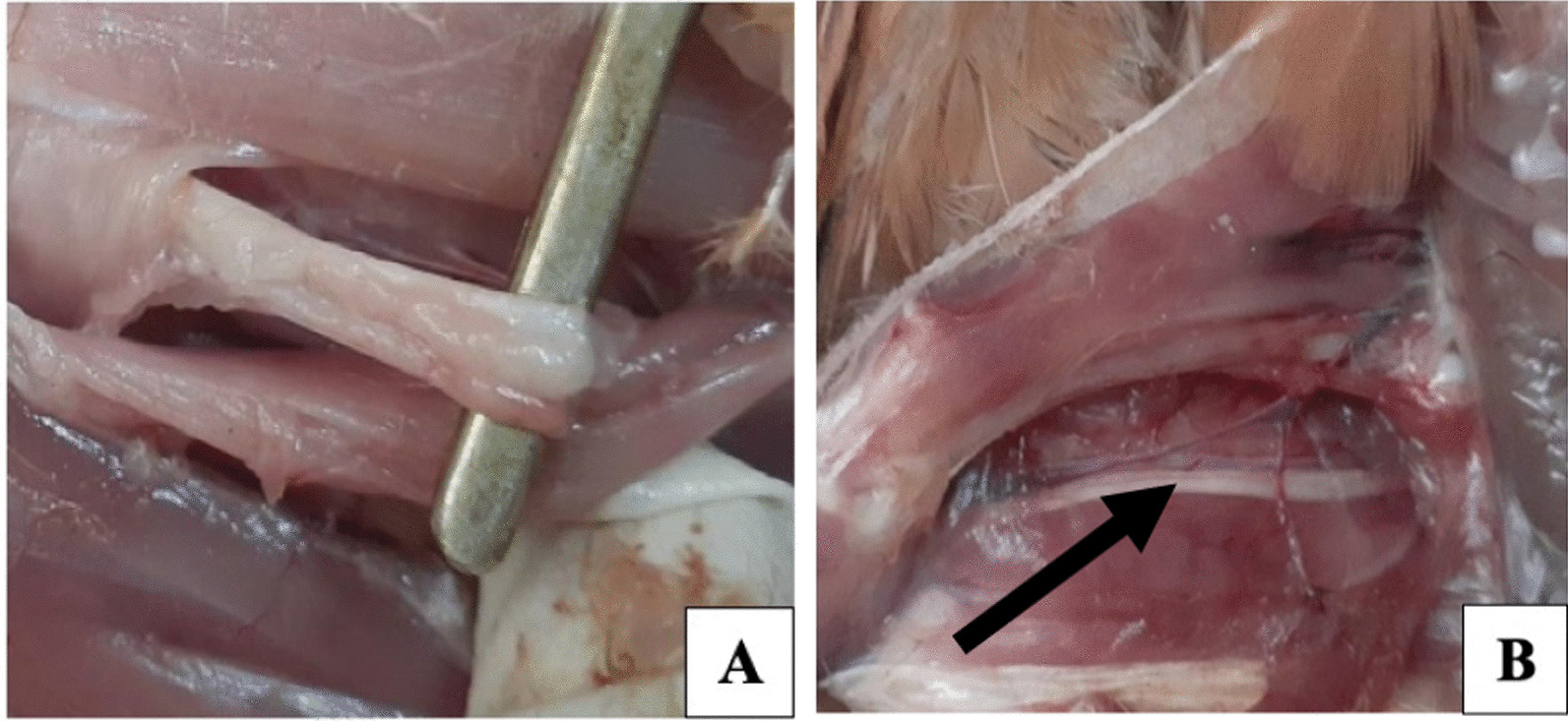
Postmortem findings of marek’s disease suspected chickens. **A** Enlarged sciatic nerve, **B** Normal sciatic nerve

Gross lesions indicatives of MD were observed on visceral organs such as spleen, liver, heart, kidney, and sciatic nerves of all chickens examined. Single or multiple greyish white to yellow tumor-like nodular lesions of various size were appreciated in visceral organs: spleen (97.14%, 68/70), liver (61.43%, 43/70), heart (47.14, 33/70), kidney (24.30%, 17/70), and sciatic nerve (84.3%, 59/70). In addition, macroscopic changes such as splenomegaly, hepatomegaly, renomegaly, and sciatic nerve enlargement were observed during necropsy examination. Follicular hyperplasia was also noticed on some of the affected birds. Of the total chickens examined, 72.86% (51/70) showed the acute visceral form, 11.43% (8/70) showed paralytic form, and 15.71% (11/70) showed the mixed form (Table 1) (Fig. 2).

**Table 1:** Summary of the gross pathological findings up on postmortem examination of MD suspected chickens

Study area	Sample	Examination method	Number of chickens examined	Number of chickens with lesions	Organs (%)
North Gondar	Spleen	Gross pathology	35	34	97.14
	Liver	Gross pathology		21	60.00
	Heart	Gross pathology		19	54.29
	Kidney	Gross pathology		11	31.43
	Sciatic nerve	Gross pathology		30	85.71
South Gondar	Spleen	Gross pathology	12	11	91.67
	Liver	Gross pathology		9	75.00
	Heart	Gross pathology		5	41.67
	Kidney	Gross pathology		4	33.33
	Sciatic nerve	Gross pathology		10	83.33
West Gojjam	Spleen	Gross pathology	23	23	100.0
	Liver	Gross pathology		13	56.52
	Heart	Gross pathology		9	39.13
	Kidney	Gross pathology		2	8.70
	Sciatic nerve	Gross pathology		19	82.61

### Marek’s disease virus isolation

**Fig. 3:**
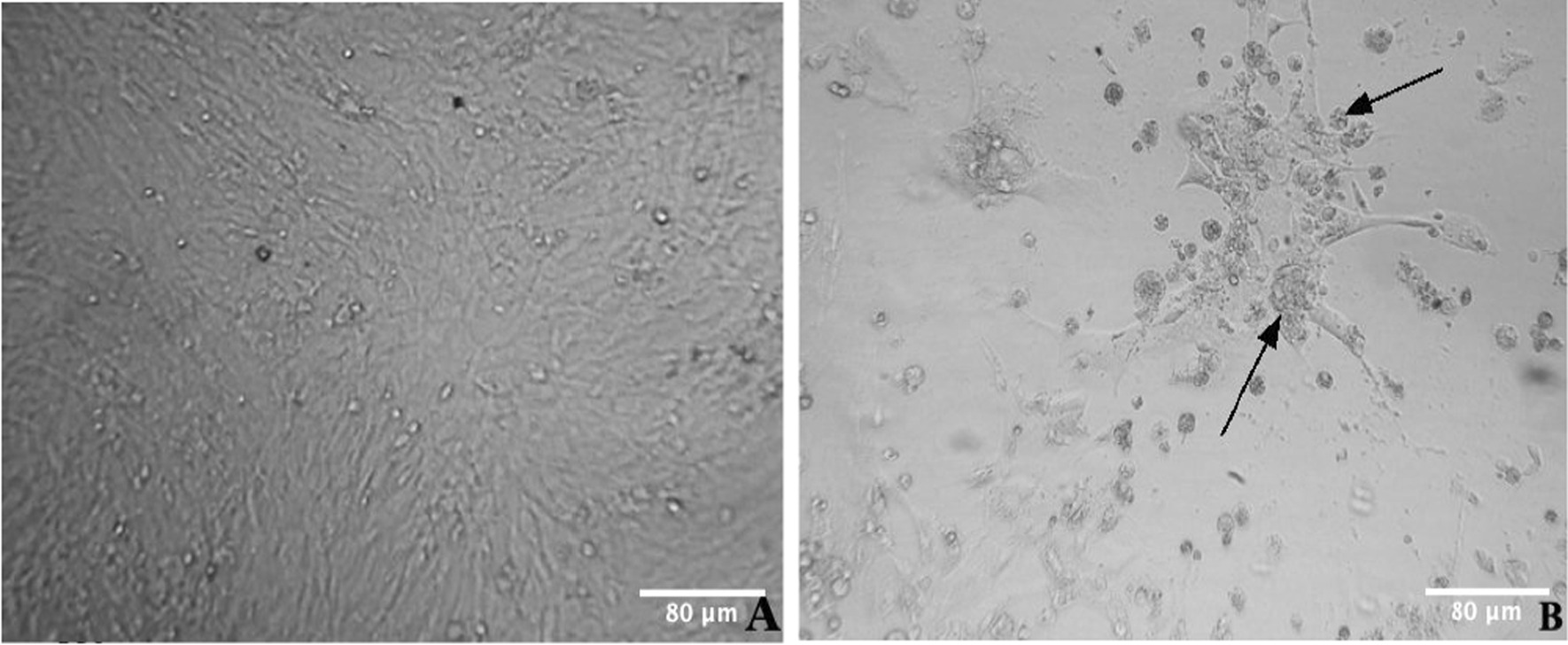
Isolation of marek’s disease virus using chicken embryo fibroblast cells. Uninfected confluent monolayer of CEF **(A)** MDV-1 infected CEF with characteristics CPEs observed after 4 days post inoculation of 2nd passage (indicated by arrows) **(B)**

Collected samples were pooled into 27 samples as shown in Table 2, and the virus was isolated from 22 preparations (81.48%). Of this, virus was supposedly isolated from 5 (71.42%) and 17 (85%) pooled spleen and feather samples respectively. Area wise, characteristic CPE was observed from 8, 7, and 7 samples from North Gondar, South Gondar and West Gojjam zones respectively (Table 2). The cytopathic effects (CPE) were visible as small plaques starting from the 4th day of the 2nd blind passages. An early CPE was observed as small round cells. These cells formed foci and syncytia that detached later from the wall of the cell culture flask causing the formation of plaque (Fig. 3).

**Table 2:** Sample collection area, number of marek’s disease-suspected tissue samples, and virus isolation rate

Study area	Sampled tissue	Test method	Number of tested samples	Number of MDV-suspected samples
North Gondar	Spleen	Cell culture	3	2 (66.66%)
	Feather	Cell culture	6	6 (100.0%)
South Gondar	Spleen	Cell culture	2	2 (100.0%)
	Feather	Cell culture	7	5 (71.43%)
West Gojjam	Spleen	Cell culture	2	1 (50.00%)
	Feather	Cell culture	7	6 (85.71%)
		**Total**	**27**	**22 (81.48%)**

Bold values indicate the distinguishability of the result

### Molecular confirmation of marek’s disease virus

**Fig. 4:**
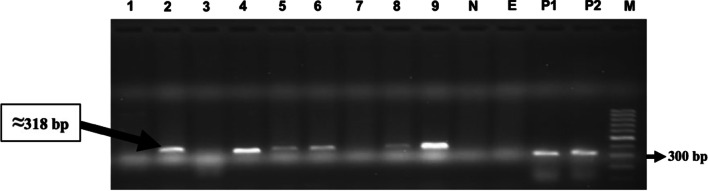
Agarose gel electrophoresis pattern of PCR amplified *ICP4* gene fragment (318 bp) of marek’s disease virus. Lane 1–9: clinical samples, *N*: Non-template control, *E*: RNase free water extraction control, and P1 and P2: Positive controls, M: 100 bp DNA ladder

Of the total 22 pooled MD-suspected clinical samples tested, 9 were found positive by PCR testing showing an approximate amplicon size of 318 bp. Some representative isolates that are positive for *ICP4* gene are shown in Fig. 4.

### Sequence analysis

**Fig. 5:**
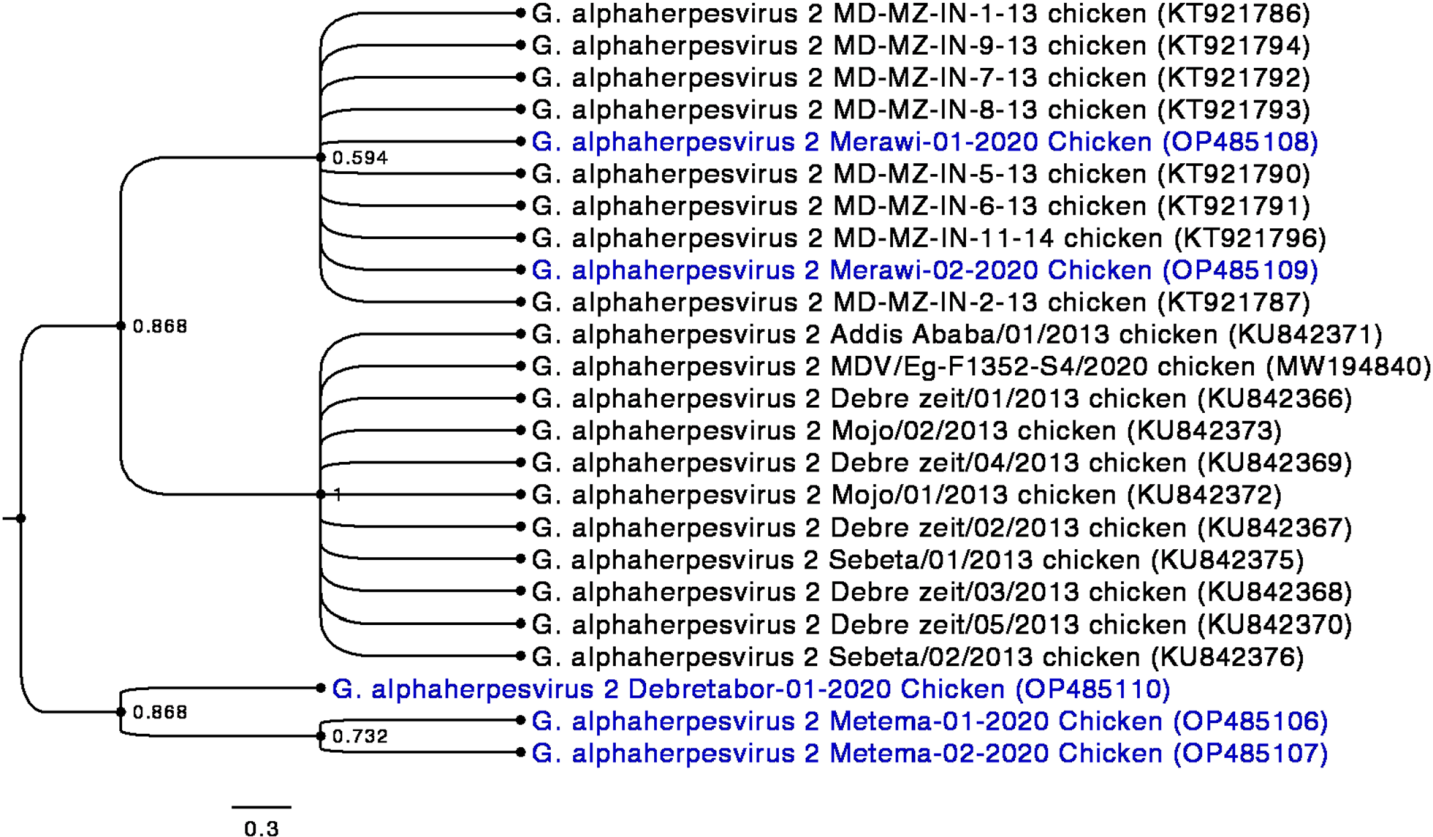
Phylogenetic relationship between 24 *marek’s disease virus* strains including the 5 strains from Northwest Ethiopia based on *ICP4* gene partial sequences using MEGA 11. The evolutionary history was inferred by using the Maximum Likelihood method and Kimura 2-parameter model [33]. The bootstrap consensus tree inferred from 1000 replicates [34] is taken to represent the evolutionary history of the taxa analyzed. Bootstrap support values are given in decimals. Codon positions included were 1st + 2nd + 3rd + Noncoding. There were a total of 401 positions in the final dataset. Taxa highlighted in blue represent MDV-1 isolates from the present study

In this study, partial region of the *ICP4* gene of five representative PCR positive isolates from each outbreak areas were sequenced successfully further confirming the identity of the virus. The *ICP4* partial gene sequences were submitted to GenBank with the accession numbers shown in Table 3. Phylogenetic analysis based on *ICP4* gene showed that MDV isolates from Northwest Ethiopia resolved into three genotypes consistent with the site of isolation. Isolates from Metema (GenBank accession numbers: OP485106 and OP485107) and Debretabor (GenBank accession number: OP485110) formed distinct clusters, the Metema isolates being phylogenetically more related to each other (Fig. 5). On the other hand, the isolates from Merawi (GenBank accession numbers: OP485108 and OP485109) clustered with 8 GaHV-2 strains from India, G. alphaherpesvirus 2 MD-MZ-IN-1–13 chicken (KT921786), G. alphaherpesvirus 2 MD-MZ-IN-2–13 chicken (KT921787), G. alphaherpesvirus 2 MD-MZ-IN-5–13 chicken (KT921790), G. alphaherpesvirus 2 MD-MZ-IN-6–13 chicken (KT921791), G. alphaherpesvirus 2 MD-MZ-IN-7–13 chicken (KT921792), G. alphaherpesvirus 2 MD-MZ-IN-8–13 chicken (KT921793), G. alphaherpesvirus 2 MD-MZ-IN-9–13 chicken (KT921794), and G. alphaherpesvirus 2 MD-MZ-IN-11–14 chicken (KT921796) (Fig. 5). Moreover, other GaHV-2 strains from Ethiopia (GenBank accession numbers: KU842366, KU842367, KU842368, KU842369, KU842370, KU842371, KU842372, KU842374, KU842375, and KU842376) and Egypt (GenBank accession number: MW194840) included in this analysis, grouped into same clade (Fig. 5).

**Table 3:** MDV isolates characterized in the present study. The MDV (*n* = 5) with isolate name, date of sample collection, and the GenBank accession number for the partial nucleotide sequences of the *ICP4* gene

S/N	Isolate name	Date of sample collection	Accession number
1	MDV/Metema/01/2020	04/24/2020	OP485106
2	MDV/Metema/02/2020	04/24/2020	OP485107
3	MDV/Merawi/01/2020	05/15/2020	OP485108
4	MDV/Merawi/02/2020	05/15/2020	OP485109
5	MDV/Debretabor/01/2020	05/26/2020	OP485110

## Discussion

In the present, outbreak-based investigation, gross pathological examination was conducted on chickens suspected of being morbid and dead of MDV infection. marek’s disease virus was isolated from tissue samples, PCR confirmed, and representative samples sequenced. This is the first confirmatory report of MDV in chicken farms located at different agro-ecological zones and production systems in Northwestern Ethiopia. Thus, the results presented here contribute to the accumulating knowledge of MD in country.

In this study, the clinical signs shown by affected chickens such as depression, shrunken combs, and paralysis of leg, wing, and neck, are consistent with literature description of MD in chickens reported elsewhere [13]. Upon postmortem examination of dead and sacrificed chickens, tumor-like nodular lesions of various size were widely observed in visceral organs. In addition, gross abnormalities such as splenomegaly, hepatomegaly, renomegaly, and sciatic nerve enlargement were recorded which were concordant with reports of other studies [14, 15].

Diagnosis of MD is usually based on isolation and identification of MDV in cell culture and identification by cytopathic changes (plaque formation) [16]. In this study, pooled spleen and feather follicle samples were targeted for virus isolation. Of the 22 samples considered positive for MDV isolation, 17 (85%) were from pooled feather samples. In fact, it has been reported that the epithelium of feather follicles is the tissue most commonly found positive in infected chickens, compared to other tissues [35, 36].

Characteristic CPEs such as rounding of cells, formation of foci area and syncytia that detached later from the wall of the cell culture flask were observed on CEF cell cultures. In line with this, other authors [27, 28] reported similar cytopathic changes on CEF upon inoculation with MD suspected tissue samples. However, CEF is permissible for other poultry herpesviruses [37, 38] that can induce similar or comparable cytopathic changes. Therefore, the identity of MDV was confirmed using a conventional PCR, amplifying 318 bp of the *ICP4* gene of MDV-1. Of the 22 pooled samples, 9 (33.33%) were found PCR-positive. Similarly, several other authors have targeted the *ICP4* gene for genetic confirmation of MDV-1 [17, 27, 39, 40]. Amplification of the conserved *ICP4* GaHV-2 gene is expected to be more reliable as the gene is specific for pathogenic MDV, MDV-1.

In order to have a better understanding of the epidemiologic situation, *ICP4* genes from 5 samples that vary by farm and study site were partially sequenced. Phylogenetic tree was re-constructed using *ICP4* partial gene sequences of the current study and homologous sequences retrieved from GenBank. Two of the isolates from the same site, Metema (GenBank accession numbers: OP485106 and OP485107) seem to be clonal complexes forming distinct cluster (bootstrap value: 73.2). The other three isolates, two from Merawi (GenBank accession numbers: OP485108 and OP485109) and one isolate from Debretabor (GenBank accession number: OP485110) seem to represent distinct genotypes although the isolate from Debretabor is closer to the Metema clonal complex (bootstrap value: 86.8). On the other hand, the isolates from Merawi appeared genetically far related to the rest of the 3 isolates and clustered with Indian MDV strains included in the analysis (bootstrap value: 59.4). Moreover, none of the isolates from the current study clustered with strains from central Ethiopia, indicating the high diversity of the virus in the country. This is consistent with previous reports of the continuous evolution of field MDV strains [30].

As mentioned before, there are 4 pathotypes of the serotype 1 MDV, mild (m), virulent (v), very virulent (vv), and very virulent + (vv +) [1]. In addition to their association with distinct virulence and subsequent losses in commercial chicken flocks, each pathotype appeared to encode various level of resistance against defined MD vaccines. For example, v pathotype strains induced high levels of disease in non-vaccinated chickens, but little disease in chickens vaccinated with HVT. In contrast, vv pathotype strains induced high levels of disease in HVT-vaccinated chickens, but little disease in chickens vaccinated with bivalent vaccines composed of HVT and selected serotype 2 strains such as SB-1 or 301B/1 [9]. Therefore, pathotyping would have a practical significance when it comes to vaccination-based control of MD. However, as a limitation, in our study, we did not conduct molecular analysis of genes that have profound impact on virulence and enable pathotyping of MDV, such as marek’s EcoRI-Q (*MEQ*), phosphoprotein-38 (*pp38*) and viral interleukin 8 (*vIL-8*) [41, 42], neither did we perform pathotypic classification of the isolates using the Avian Disease and Oncology Laboratory (ADOL) method.

Based on the comparative phylogenetic analysis, it can be speculated that the virus is introduced to the country through importation of vaccines, chickens’ eggs or live chickens. Despite the true sources of the virus, it has become clear that MDV is circulating in chicken farms in Northwest Ethiopia. The current live chicken market and the extensive movement of chickens within and across the region coupled with lack of MD preventive measures, is expected to favor the spread of the virus which will result in a severe consequence.

## Conclusion

Information on the epidemiology of MD in Ethiopia has been increasing over the past few years. This study presented the first molecular evidence of MDV in chicken farms from Northwest Ethiopia. The findings of this study are potential indicators of the circulation of MDV in chicken farms in the study areas. Given the lack of strict control and preventive measures against MD in the country, further outbreaks of the disease could strike easily and widely resulting in serious harm to the poultry sector. Biosecurity measures should strictly be implemented to hinder the spread of the virus and vaccines selection should be dictated by determination of pathotype of the field MDV-1 isolates by molecular tools. Nationwide studies on molecular characteristics of MDV isolates, their pathotypes, and estimation of the economic impact associated with the disease may help justify production and use of MD vaccines within the country.

## Data Availability

All data generated or analysed during this study are included in this article and its supplementary information file.
